# Effect of Different Nursing Interventions on Discharged Patients with Cardiac Valve Replacement Evaluated by Deep Learning Algorithm-Based MRI Information

**DOI:** 10.1155/2022/6331206

**Published:** 2022-03-21

**Authors:** Jing Zhang, Qiong Zhou

**Affiliations:** ^1^Department of Cardiology Second Ward, Jingzhou First People's Hospital, No. 8 Hangkang Road, Jingzhou, Hubei Province 434000, China; ^2^Department of Cardiology Third Ward, Jingzhou First People's Hospital, No. 8 Hangkang Road, Jingzhou, Hubei Province 434000, China

## Abstract

This study was aimed to explore the application of cardiac magnetic resonance imaging (MRI) image segmentation model based on U-Net in the diagnosis of a valvular heart disease. The effect of continuous nursing on the survival of discharged patients with cardiac valve replacement was analyzed in this study. In this study, the filling completion operation, cross entropy loss function, and guidance unit were introduced and optimized based on the U-Net network. The heart MRI image segmentation model ML-Net was established. We compared the Dice, Hausdorff distance (HD), and percentage of area difference (PAD) values between ML-Net and other algorithms. The MRI image features of 82 patients with valvular heart disease who underwent cardiac valve replacement were analyzed. According to different nursing methods, they were randomly divided into the control group (routine nursing) and the intervention group (continuous nursing), with 41 cases in each group. The Glasgow Outcome Scale (GOS) score and the Self-rating Anxiety Scale (SAS) were compared between the two groups to assess the degree of anxiety of patients and the survival status at 6 months, 1 year, 2 years, and 3 years after discharge. The results showed that the Dice coefficient, HD, and PAD of the ML-Net algorithm were (0.896 ± 0.071), (5.66 ± 0.45) mm, and (15.34 ± 1.22) %, respectively. The Dice, HD, and PAD values of the ML-Net algorithm were all statistically different from those of the convolutional neural networks (CNN), fully convolutional networks (FCN), SegNet, and U-Net algorithms (*P* < 0.05). Atrial, ventricular, and aortic abnormalities can be seen in MRI images of patients with valvular heart disease. The cardiac blood flow signal will also be abnormal. The GOS score of the intervention group was significantly higher than that of the control group (*P* < 0.01). The SAS score was lower than that of the control group (*P* < 0.05). The survival rates of patients with valvular heart disease at 6 months, 1 year, 2 years, and 3 years after discharge were significantly higher than those in the control group (*P* < 0.05). The abovementioned results showed that an effective segmentation model for cardiac MRI images was established in this study. Continuous nursing played an important role in the postoperative recovery of discharged patients after cardiac valve replacement. This study provided a reference value for the diagnosis and prognosis of valvular heart disease.

## 1. Introduction

Cardiac valve disease is a common cardiovascular disease with abnormal cardiac valve structure or function. There are about 15 million patients with cardiac valve disease worldwide, accounting for 50% of cardiovascular diseases [1]. Cardiac valve disease causes hemodynamic changes in patients, which eventually lead to heart failure, arrhythmia, embolism, and other complications [2]. Cardiac valve replacement is a common treatment for valvular heart disease, but long-term anticoagulation therapy is required after surgery [3]. Due to the limited awareness of the disease, thrombosis may occur due to lack of anticoagulant effect or bleeding due to excessive anticoagulant effect. Finally, the effects of the operation and the quality of life of patients are affected [4]. Continuous nursing is a kind of continuous nursing for patients after discharge under the guidance of medical staff to reduce the occurrence of postoperative adverse symptoms and improve the quality of life after operation [5]. Some studies showed that quality care for patients after heart valve replacement can significantly reduce the incidence of postoperative complications. Moreover, the guidance of professional nursing staff can reduce the incidence of adverse reactions in anticoagulant therapy and improve the prognosis of patients [6]. However, most studies were studying the influence of nursing methods on patients during hospitalization, and there were few studies on the influence of nursing methods on patients after discharge.

Ultrasound cardiogram (UCG) is a common imaging method in the diagnosis of valvular heart disease. UCG has the characteristics of good accuracy and being cost-effective, which is the gold standard for the diagnosis of valvular heart disease. However, it can only be semiquantitatively analyzed in the evaluation of differential pressure and reverse flow [7]. MRI can accurately quantify the differential pressure and reverse flow in the diagnosis of heart valve disease. MRI can accurately quantify the differential pressure and reverse flow in the diagnosis of heart valve disease. However, at present, most of the current cardiac MRI images are mostly divided manually, which has problems such as subjectivity, poor repeatability, and time-consuming. In addition, quantitative diagnosis of cardiac diseases is carried out [8]. Some researchers applied deep learning algorithms to MRI image segmentation. However, the heart structure is complex and the tissue boundary is blurred. And the images captured by the heart beat produced motion artifacts and noise [9]. These factors have brought great challenges to the accurate positioning and segmentation of cardiac structures. The U-Net in deep learning can obtain higher segmentation accuracy with less training times. Some researchers established a heart segmentation method based on the U-Net network with faster training speed and less memory occupation [10]. However, in the application process, it is found that due to uncontrollable factors in the process of MRI image acquisition, the distribution of image pixel values is different and the image data have problems such as noise. Directly using the original image leads to a certain deviation in segmentation [11]. Therefore, it needs to be further optimized.

In summary, there are few studies on the postdischarge care of patients after cardiac valve replacement. MRI technology still has the phenomenon of segmentation result deviation in the diagnosis process. The U-Net was optimized to increase its accuracy in MRI image segmentation, which was then applied to the diagnosis of heart valvular disease. Continuous nursing was used in patients after cardiac valve replacement to explore the impact of continuous nursing on postoperative quality of life. It was hoped to provide a reference value for the diagnosis and prognosis of clinical heart valvular disease.

## 2. Materials and Methods

### 2.1. Region of Interest (ROI) Detection Based on the U-Net Network

In order to increase the accuracy of MRI image segmentation, MRI images need to be preprocessed before ROI detection using the U-Net network. It mainly includes three parts, which are as follows: data normalization processing, image denoising, and image normalization. The data normalization is mainly normalizing the original image to the same size, so that each image voxel size is as consistent as possible [12]. ReLU is used as the activation function in the middle layer of a convolutional neural network. The calculation method of ReLU is expressed as follows:

(1) $$\begin{array}{c} G\left(X\right)=\max\left(0,X\right). \end{array}$$

The input MRI image is mapped to (0, ∞), and the final output layer uses softmax activation function. The calculation method is expressed as follows:

(2) $$\begin{array}{c} G_{i}\left(Y\right)=\frac{e^{zi}}{\sum_{j\in \text{group}}e^{zj}}. \end{array}$$

The output probability is (0, 1), so the network input of image MRI after processing is normalized (0, 1).

Originally acquired MRI images include parts of the heart and organs around the heart. In order to ensure the accuracy of the whole heart structure segmentation network, the original labels need to be preprocessed. Its specific processing method can be expressed as follows:

(3) $$\begin{array}{c} L\left(i,j,k\right)=\left\{\begin{array}{cc} 0, & d\left(i,j,k\right)=b,\\ 1, & d\left(i,j,k\right)=s_{m},\;m=1,2,L7. \end{array}\right. \end{array}$$

In the equation, *b* is the background label value in the original label image, and *s*_*m*_ is the label value of the seven-seed structure of the heart in the original label image. They are the left ventricular chamber (LV), right ventricular chamber (RV), left atrial chamber (LA), right atrial chamber (RA), left ventricular myocardium (Myo), ascending aorta (aorta), and pulmonary artery (PA).

**Figure 1:**
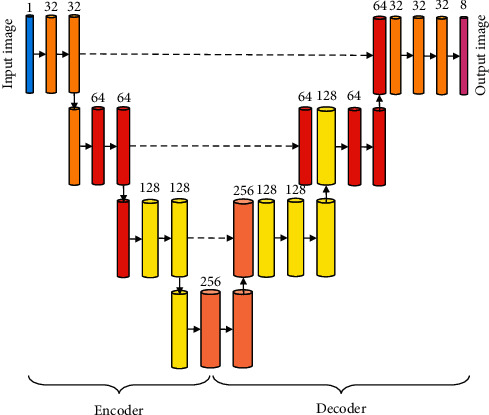
ROI detection structure based on the U-Net network.

The main body of the U-Net network is composed of encoding and decoding structures. Coding and decoding structures play an important role in image processing. The convolution layer and pooling layer can be used to obtain the image feature map in MRI image processing [13]. Decoding is converting the feature map into the feature map required for a specific task through the convolution layer and the transposition convolution layer [14]. The encoding and decoding structure based on the U-Net network can ensure the reuse of feature maps of the same size and reduce the loss of information during the pooling process in the encoding phase [15]. In this study, the input and output sizes of the basic convolution unit were consistent throughout the filling and completion operations. The ROI detection structure based on the U-NET network is shown in Figure 1.

The last layer of the network uses the cross-entropy function and softmax. The cross-entropy function can be expressed as follows:

(4) $$\begin{array}{c} E=\sum_{x\in \Omega }w\left(x\right)\log\left[p_{l\left(x\right)}\left(x\right)\right], \end{array}$$

in the equation, *w*(*x*) is the image weight, and its calculation method is expressed as follows:

(5) $$\begin{array}{c} w\left(x\right)=w_{a}\left(x\right)+w_{0}•\;\exp\left\{\frac{{\left[d_{1}\left(x\right)+d_{2}\left(x\right)\right]}^{2}}{2\beta^{2}}\right\}, \end{array}$$

in the equation, *w*_*a*_ is used to balance the weight map of category frequency. *d*_1_ represents the distance to the nearest cell boundary. *d*_2_ represents the distance to the second nearest cell boundary. *β* is the network weight by Gaussian distribution parameters.

The loss function can show the difference between the target area segmented by the model and the actual target area [16]. Based on the classification objective of this study, the cross-entropy loss function was used as the model loss function. The calculation method of the cross-entropy loss function can be expressed as follows:

(6) $$\begin{array}{c} J\left(\delta \right)=-\frac{1}{m}\sum_{i=1}^{m}\left\{y^{\left(i\right)}\log\left[h_{\delta }\left(x^{\left(i\right)}\right)\right]+\left(1-y^{\left(i\right)}\right)\log\left[1-h_{\delta }\left(x^{\left(i\right)}\right)\right]\right\}, \end{array}$$

in the equation, *m* denotes the number of categories. *n* is the number of voxels. *y*^(*i*)^ is the true probability of the *i*-prime being class *m*. *x*^(*i*)^ is the prediction probability of the *i*-prime being class *m*.

**Figure 2:**
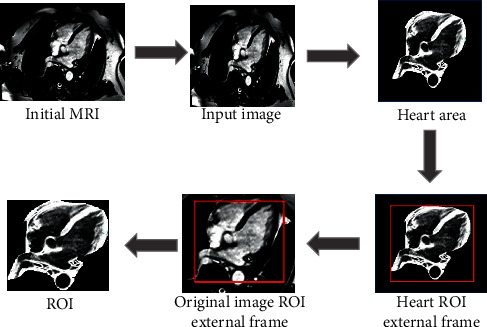
ROI detection process based on the U-NET network.

The original image is adjusted to a size suitable for the U-Net network inspection. The adjusted MRI image is input into the optimized U-Net network for heart segmentation. The external cube frame (the red boxes in Figure 2) of the heart region is calculated based on the preliminary heart segmentation results, and it is used as the initial ROI of the heart image. Then, it expands five voxels outward to restore the original image to obtain the final original image. Then, the ROI detection results of the original image are obtained. The ROI detection process based on the U-NET network is shown in Figure 2.

### 2.2. MRI Image Segmentation Model Based on the U-Net Network

**Figure 3:**
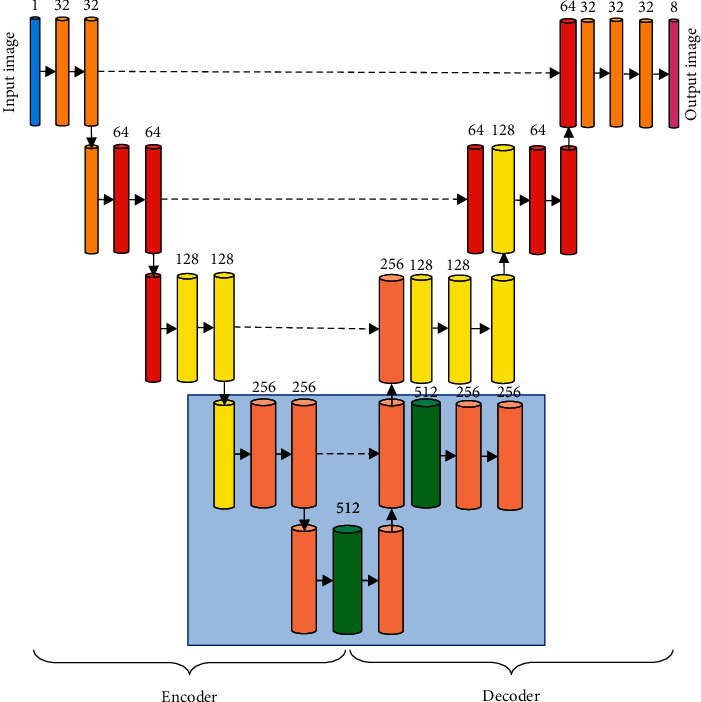
ML-Net heart segmentation network structure diagram.

Cardiac MRI images were further segmented by ROI detection results based on the U-Net network in this study. The U-Net networks typically update parameter weights through the last layer of output calculation errors. In the process of operation, due to the deep network, it led to a lack of guidance for the updating of low-level network parameters. In this study, each layer on the U-Net network expansion path was used as a label prediction with a certain resolution. Multiple outputs of different scales were increased on the expansion path. The guidance unit was introduced to establish a new heart MRI image segmentation method, and it was named ML-Net. The guidance unit can guide the high-level segmentation through the features extracted at the low level to increase the accuracy of image segmentation. Therefore, the higher the output, the finer the prediction results. The ML-Net heart segmentation network structure is shown in Figure 3.

The loss function of the ML-Net heart segmentation network is calculated based on the Dice coefficient. The calculation method is expressed as follows:

(7) $$\begin{array}{c} J\left(\delta \right)=1-\frac{2\times \left|y\cap g_{\delta }\left(x\right)\right|}{\left|y\cup g_{\delta }\left(x\right)\right|}. \end{array}$$

In the equation, *y* is the real category of all voxels, and *g*_*δ*_(*x*) is the predicted category of all voxels.

### 2.3. Performance Evaluation of MRI Image Segmentation

In this study, Dice coefficient, Hausdorff distance (HD), and percentage of area difference (PAD) were used as the evaluation indexes of model segmentation effect. The Dice coefficient measures the proximity between the target real area and the predicted area. The closer the Dice coefficient is to 1, the better the model segmentation performance is. The calculation method is expressed as follows:

(8) $$\begin{array}{c} \text{Dice}=\frac{2\left|C\cap D\right|}{\left|C\right|+\left|D\right|}. \end{array}$$

HD is the symmetric distance measure of the maximum difference between the two contours. The smaller the HD value, the better the segmentation performance. The calculation method is expressed as follows:

(9) $$\begin{array}{c} H\;D\left(C,D\right)=\underset{a\in C}{\max}\left\{\underset{a\in D}{\max}\left[s\left(a,b\right)\right]\right\}. \end{array}$$

PAD is also an evaluation index for medical image segmentation. The smaller the PAD value, the better the model segmentation performance. The calculation method is expressed as follows:

(10) $$\begin{array}{c} \text{PAD}=\frac{\left|C-D\right|}{C}. \end{array}$$

In equations (8)∼(10), *s*(*a*, *b*) is the Euclidean distance between two points *a* and *b*. *C* is the predictive output graph of the model. *D* is the gold standard drawn manually.

### 2.4. Research Subject and Grouping

A total of 82 patients who underwent cardiac valve replacement in hospital from December 2017 to June 2021 were selected as the study subjects. All patients underwent MRI examinations. There were 50 males and 32 females. The age of the patients ranged from 33 to 71 years. The average age was 50.64 ± 6.89 years. The inclusion criteria were as follows: (1) patients clinically diagnosed as rheumatic heart disease; (2) patients who underwent cardiac valve replacement for the first time; (3) patients with cardiac function of grade I–III; and (4) patients with hemiplegia, cerebral infarction, pulmonary function, and other diseases. The exclusion criteria were as follows: (1) patients with communication or disturbance of consciousness; (2) patients with severe liver and kidney dysfunction or other cancers; and (3) MRI images were not clear and did not meet the clinical application standards. All subjects were randomly divided into the control group (routine nursing) and the intervention group (continuous nursing) according to different nursing methods, with 41 cases in each group. In the conventional nursing group, there were 26 males and 15 females, ranging from 33 to 70 years old, with an average age of 50.87 ± 4.48 years old. There were 19 patients with grade I, 14 patients with grade II, and 8 patients with grade III cardiac function. In the continuous care group, there were 24 males and 17 females, ranging from 34 to 71 years old, with an average age of 49.93 ± 5.64 years old. There were 21 patients with grade I, 13 patients with grade II, and 7 patients with grade III cardiac function. There was no significant difference in age, sex ratio, and proportion of patients with different cardiac function grades between the two groups (*P* > 0.05). This study had been approved by the ethics committee of the hospital, and the subjects included in the study had signed the informed consent form.

### 2.5. MRI Examination Methods

All subjects were diagnosed using the superconducting MRI scanner with total image matrix (TIM) technology. The phase velocity coding film in the plane parallel to the blood flow direction was used to determine the blood flow state. The scanning parameters were as follows: repetition time (TR) was 65 ms, echo time (TE) was 2.8 ms, and deflection angle (FA) was 30°. A quantitative evaluation of the blood flow profile in the abdominal aorta with phase-coded cine-MR (VEC-MR) was performed. The scanning parameters were as follows: repetition time (TR) was 64 ms, echo time (TE) was 2.8 ms, and deflection angle (FA) was 30°. The scanning plane was set at the fastest flow rate or valve level, and the phase diagram of 20–25 frames in one cardiac cycle can be obtained by one scan.

Blood flow analysis software was used to manually analyze the blood flow phase map. The blood flow parameters such as maximum velocity, forward blood flow, unit time flow, average flow, average velocity, and cross-sectional area of blood flow were calculated in one cardiac cycle. According to the blood flow phase diagram, the forward flow and reverse flow of each cardiac cycle were obtained. The reflux index was calculated, 15%–20% for mild, 20%–40% for moderate, and more than 40% for severe.

### 2.6. Nursing Intervention Methods and Observation Indicators

The control group was given routine nursing care; that is, the nursing staff of the department gave guidance to the patients after discharge. The main contents included diet, medication, activities, and other matters needing attention. It was recommended that patients receive regular reviews. The intervention group was given continuous nursing intervention based on routine nursing in the control group. After patient discharge, full-time nursing staff regularly conducted family visits or telephone inquiries, and the frequency was 1 time/month. A systematic and standardized continuous nursing evaluation scale was developed with sleep quality, drug dosage, daily diet content, heart rate, and blood pressure changes as the main guidance content. Full-time nursing staff provided healthy diets for patients. Patients were instructed to perform regular wound reexamination, blood tests, MRIs, and electrocardiogram (ECG) examinations. The drug dosage was adjusted according to the test results.

The Glasgow Outcome Scale (GOS) was used to evaluate the prognosis of patients. 1 point represented death, 2 points represented severe disability, 3 points represented inability to take care of themselves, 4 points represented independent living, and 5 points represented normal living [17]. The Self-rating Anxiety Scale (SAS) was used to evaluate the anxiety degree of patients. The higher the SAS score, the more serious the anxiety [18]. The survival of the patients at 6 months, 1 year, 2 years, and 3 years after discharge was counted.

### 2.7. Statistical Methods

The experimental data were processed by SPSS19.0 statistical software. The measurement data were expressed as the mean ± standard deviation (x(−) ±*s*), the count data were expressed as percentage (%). The *χ*^2^ test was used. *P* < 0.05 indicated that the difference was statistically significant.

## 3. Results

### 3.1. The ML-Net Heart Segmentation Results Analysis

**Figure 4:**
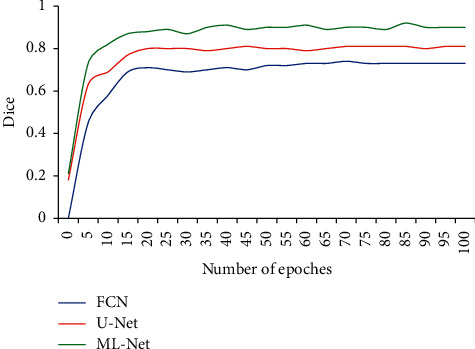
Curves of Dice values of different algorithms changing with iterations.

Under the same training set, the influence of iteration times of the FCN, U-NET, and ML-NET on Dice value in the heart segmentation process was verified (Figure 4). As the number of iterations increased, the Dice values of FCN, U-Net, and ML-Net algorithms increased first and then tended to be stable. At the same iteration number, the Dice value of the ML-Net network was higher than that of the FCN and U-Net.

**Figure 5:**
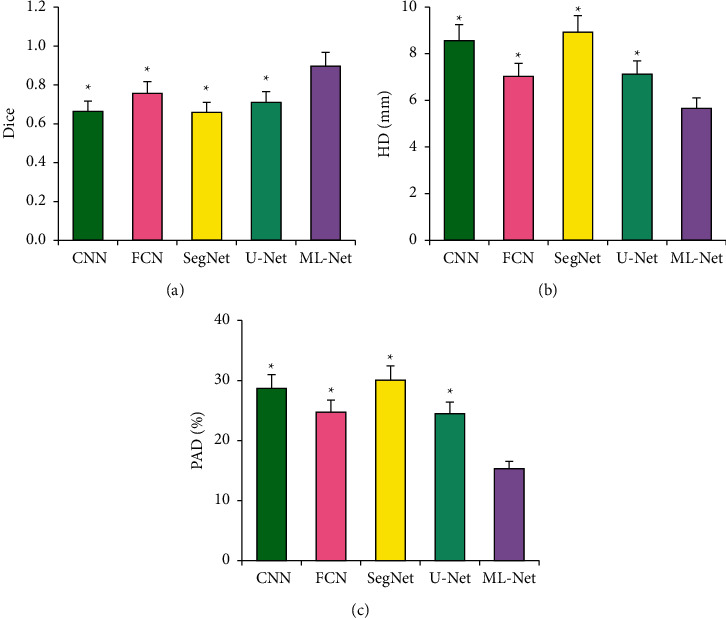
Analysis of heart segmentation results of different algorithms. (a) Dice coefficient comparison; (b) HD value comparison; (c) PAD value comparison.  ^*∗*^ Compared with the ML-Net algorithm, (*P* < 0.05).

The Dice, HD, and PAD values of heart segmentation by CNN, FCN, SegNet, U-Net, and ML-Net algorithms were compared and analyzed (Figure 5). The maximum Dice coefficient of the ML-Net algorithm was (0.896 ± 0.071). HD and PAD were the smallest, which were (5.66 ± 0.45) mm and (15.34 ± 1.22) %, respectively. The Dice, HD, and PAD values of the ML-Net algorithm were statistically different from those of other algorithms (*P* < 0.05).

### 3.2. MRI Features of Valvular Heart Disease

**Figure 6:**
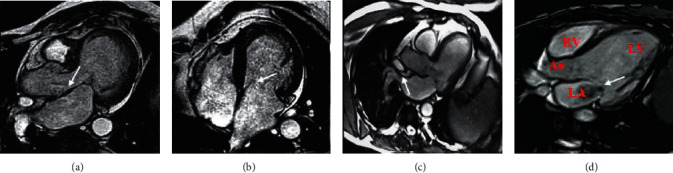
(a) Patient with severe mitral valve disease, 61 years old, male. Left atrial height, enlargement, aortic stenosis; (b) a 54-year-old male patient with mitral insufficiency caused by hypertrophic cardiomyopathy. Left atrial enlargement; (c) patient with mitral insufficiency due to left ventricular dysfunction, 47 years old. Left atrium and left ventricle increased; (d) in patients with severe mitral valve disease, the left atrial (LA) and left ventricular (LV) cavities were significantly enlarged during the left ventricular systolic phase of the four-chamber view of the heart.

MRI images of patients with valvular heart disease showed abnormalities in the atrium, ventricle, and main artery. And the patient's cardiac blood flow signal was abnormal. Aortic stenosis can be seen in patients with severe mitral valve disease (shown by the arrow), and the left ventricular wall was concentrically thickened (Figure 6(a)). Patients with mitral insufficiency caused by hypertrophic cardiomyopathy mainly showed left atrial enlargement (shown by the arrow), and the left ventricular cavity was not significantly increased (Figure 6(b)). MRI images of patients with mitral insufficiency caused by left ventricular dysfunction showed enlargement of left atrium and left ventricular cavity (shown by arrow) (Figure 6(c)). In patients with severe mitral valve disease, the left atrium near the mitral valve orifice during the left ventricular systolic period showed obvious reflux of high-speed blood flow signals (shown by the arrow in Figure 6(d)).

### 3.3. Analysis of MRI Results

**Figure 7:**
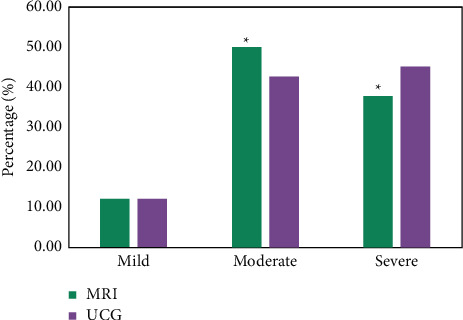
Comparison of MRI and UCG in the diagnosis of Cardiac Valvular Regurgitation Index. *∗* Compared to UCG, (*P* < 0.05).

The diagnostic performance of MRI for valvular heart disease was analyzed using echocardiography (UCG) as the gold standard (Figure 7). The proportion of patients with mild, moderate, and severe reflux indexes detected by UCG was 12.20%, 42.68%, and 45.12%, respectively. The proportion of patients with mild, moderate, and severe reflux indexes detected by MRI was 12.20%, 50.00%, and 37.80%, respectively. There was statistical difference between them (*P* < 0.05).

### 3.4. Analysis of Nursing Results of Cardiac Valve Replacement

**Figure 8:**
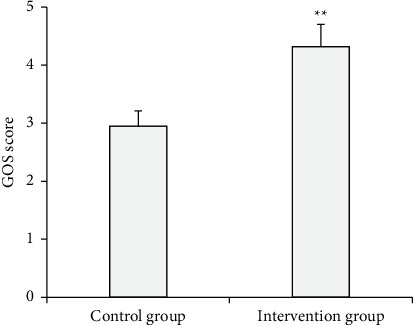
Comparison of the GOS scores of different nursing methods.  ^*∗∗*^ Compared with the control group, (*P* < 0.01).

The GOS scores of the control group and the intervention group after intervention were compared and analyzed (Figure 8). The results showed that the GOS score of the intervention group was significantly higher than that of the control group (*P* < 0.01).

**Figure 9:**
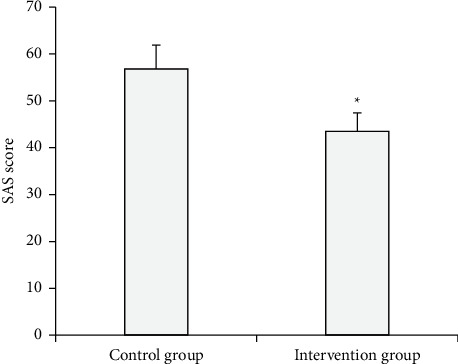
Comparison of the SAS scores of different nursing method.  ^*∗*^ Compared with the control group (*P* < 0.05).

The SAS scores of the control group and the intervention group after intervention were compared and analyzed (Figure 9). The results showed that the SAS score of the intervention group was lower than that of the control group (*P* < 0.05).

**Figure 10:**
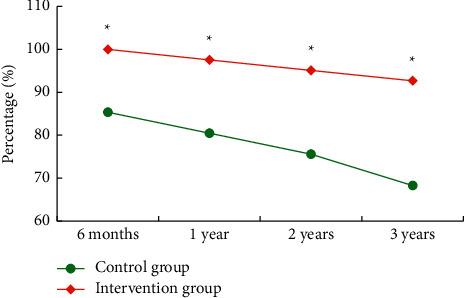
Comparison of the survival rate of different nursing methods.  ^*∗*^ Compared with the control group (*P* < 0.05).

The survival conditions of patients in the control group and the intervention group at different times after intervention were compared and analyzed (Figure 10). The results revealed that the survival rates of 6 months, 1 year, 2 years, and 3 years after discharge in the intervention group were significantly higher than those in the control group (*P* < 0.05).

## 4. Discussion

In this study, cardiac MRI images were preprocessed based on the U-Net network to make their data distribution more consistent and meet the data requirements of neural network training [19]. The results showed that the Dice value of the ML-Net network was higher than that of the FCN and U-Net under the same iteration number. Under the same number of iterations, the Dice value of the ML-Net network was higher than that of the FCN and U-Net. The Dice coefficient of the ML-Net algorithm was the largest (0.896 ± 0.071), and its HD and PAD were the smallest, which were (5.66 ± 0.45) mm and (15.34 ± 1.22) %, respectively. Compared with other algorithms, the Dice, HD, and PAD values of the ML-Net algorithm were all statistically different (*P* < 0.05). The segmentation accuracy of the ML-Net network obtained by the optimization of the U-Net network was significantly improved. The reason was that the guidance unit in the ML-Net network sampled the low-resolution output, which enhanced the network feature extraction ability [20]. Finally, the segmentation accuracy was significantly improved. Lee et al. (2021) [21] established a brain MRI segmentation algorithm based on the U-Net algorithm and found that the Dice value of the segmented MRI image was 0.93. The Dice value of the image segmented by the established method was significantly smaller than that in this study, and the reason may be that the segmentation parts of the two were different, and the large amount of tissue around the heart had a certain impact on the segmentation, resulting in a smaller Dice value. However, the Dice value of the established model was significantly higher than that of the ventricle segmentation method established by the researchers of Zhao et al. (2020) [22] based on the U-Net algorithm.

The blood flow sensitive MR technology in MRI diagnosis played an important role in the diagnosis of cardiac valve disease [23]. The results of this study showed that there was a statistically significant difference between patients with mild, moderate, and severe UCG detection reflux index and MRI (*P* < 0.05). The results showed that the proportion of patients with a moderate reflux index detected by MRI was higher than that of UCG. Its diagnostic accuracy was obviously higher than UCG. This was consistent with the research results of Francone et al. (2020) [24]. Anticoagulant drugs should be taken for life after heart valve replacement [25]. It was unable to ensure the effective use of anticoagulant drugs for some patients due to a lack of professional nursing knowledge after discharge, resulting in poor postoperative therapeutic effect and quality of life [26]. The results revealed that the GOS score of the intervention group was significantly higher than that of the control group (*P* < 0.01). The SAS score of the intervention group was lower than that of the control group (*P* < 0.05). Continuous nursing can effectively improve the psychological state of patients. Continuing care took patients who had been controlled and needed reasonable rehabilitation nursing as the research object and provided out-of-hospital nursing guidance for patients [27]. It can effectively alleviate the adverse emotions of patients and effectively improve the postoperative quality of life of patients.

## 5. Conclusion

A heart MRI image segmentation model ML-Net was constructed based on the U-Net network, and the influence of continuous nursing on discharged patients after cardiac valve replacement was analyzed. It was found that the ML-Net could effectively improve the segmentation accuracy of cardiac MRI images. Continuous nursing can effectively improve the psychological status of discharged patients with cardiac valve replacement and improve the postoperative survival rate. However, there were still some shortcomings in this study, and the quality of life of patients with continuous nursing was not analyzed. In the future, the influence of continuous nursing on the quality of life of patients will be further analyzed. In summary, this study established an effective cardiac MRI image segmentation model. Continuous nursing played an important role in the postoperative recovery of discharged patients after cardiac valve replacement. This theory provided a reference value for the diagnosis and prognosis of valvular heart disease.

## Data Availability

The data used to support the findings of this study are available from the corresponding author upon request.
